# A Co-Nanoparticles Modified Electrode for On-Site and Rapid Phosphate Detection in Hydroponic Solutions

**DOI:** 10.3390/s21010299

**Published:** 2021-01-05

**Authors:** Feng Xu, Peng Wang, Shiyuan Bian, Yuliang Wei, Deyi Kong, Huanqin Wang

**Affiliations:** 1 Institute of Intelligent Machines, Hefei Institutes of Physical Science, Chinese Academy of Sciences, Hefei 230031, Anhui, China; xf1992@mail.ustc.edu.cn (F.X.); sybian@iim.ac.cn (S.B.); wyl1010@mail.ustc.edu.cn (Y.W.); hqwang@iim.ac.cn (H.W.); 2 Science Island Branch of Graduate School, University of Science and Technology of China, Hefei 230009, Anhui, China; 3 Department of Mechanical and Nuclear Engineering, Virginia Commonwealth University, Richmond, VA 23284, USA; wangp7@vcu.edu

**Keywords:** phosphate, buffer-free sensor, Co-nanoparticles, on-site and rapid, hydroponic solutions

## Abstract

Conventional strategies for determining phosphate concentration is limited in efficiency due to the cost, time, and labor that is required in laboratory analysis. Therefore, an on-site and rapid detection sensor for phosphate is urgently needed to characterize phosphate variability in a hydroponic system. Cobalt (Co) is a highly sensitive metal that has shown a selectivity towards phosphate to a certain extent. A disposable phosphate sensor based on the screen-printed electrode (SPE) was developed to exploit the advantages of Co-nanoparticles. A support vector machine regression model was established to predict the concentration of phosphate in the hydroponic solutions. The results showed that Co-nanoparticles improve the detection limit of the sensor in the initial state. Meanwhile, the corrosion of Co-nanoparticles leads to a serious time-drift and instability of the electrodes. On the other hand, the coefficient of variation of the disposable phosphate detection chip is 0.4992%, the sensitivity is 33 mV/decade, and the linear range is 10^−1^–10^−4.56^ mol/L. The R^2^ and mean square error of the buffer-free sensor in the hydroponic solution are 0.9792 and 0.4936, respectively. In summary, the SPE modified by the Co-nanoparticles is a promising low-cost sensor for on-site and rapid measurement of the phosphate concentration in hydroponic solutions.

## 1. Introduction

Phosphate is one of the main nutrients playing many key roles in the human body and plant growth. For crops, phosphorus is an indispensable element in the formation of nuclear protein and lecithin. Phosphorus can accelerate cell division, accelerate root and shoot growth, promote flower bud differentiation, and improve fruit quality [1,2,3,4]. However, excessive phosphorus also brings many disadvantages to plant growth and development, such as early maturity of crops, small grains, and low yield [5]. Thus, the concentration of phosphate needs to be regulated to a balanced and suitable range set, especially in the hydroponic system, all the phosphorus of plants comes from phosphate. The common rapid detection methods of phosphorus content in water can be divide into two categories as follows.

The first, optical methods, e.g., spectrophotometry, and colorimetry. Spectrophotometry can be used to detect the concentration of phosphate. The first step is to form a photoactive derivative of phosphate by reaction, then diagnose the content of the derivative by UV-Vis or other spectroscopic techniques, and finally calculate the concentration of phosphate. Colorimetry is an old and traditional method for analyzing substance content, which is called the standard technology of phosphate detection. Modern colorimetry and spectrophotometry have an inseparable relationship. For example, after the complexation of phosphate and molybdenum, the concentration of phosphomolybdate needs to be detected by the spectrometer [6]. Abbas et al. demonstrated the determination of trace amounts of phosphate ions in water by spectrophotometric and colorimetric method respectively (detection limits: 5 ng/mL and 1.66 µg/L), the polyurethane foam is used as adsorbent [7,8]. Based on the optical method, the influence of sampling conditions and methods on phosphorus detection was investigated [9,10,11], and the detection method and optimization of optical method for phosphate in freshwater [12] and drinking water [13] were studied. Pinyorospathum et al. proposed an inexpensive colorimetric sensor for the detection of phosphate ions (Pi) performed on paper-based analytical devices based on the anti-aggregation of 2-mercaptoethanesulfonate (MS)-modified silver nanoplates. This method can detect Pi in the range of 1–30 mg/L, with a detection limit of 0.33 mg/L and a limit of quantification equal to 1.01 mg/L [14]. As we known, one of the advantages of optical methods is that the sensitive element can show a high level of sensitivity and selectivity while the sensitive element does not need to contact with the solution, which reduces the possibility of measurement error due to the weakening of sensor performance. There is no doubt that there are more accurate and stable methods in the laboratory, such as ion chromatography and fluorescence method, which are not suitable for on-site detection. Although these methods have many advantages in phosphate detection, they still face major disadvantages of on-site measurement applications, including the need for complex and expensive instruments and chemical reagents, as well as the need for trained operators to measure in the laboratory environment, which makes them both expensive and impractical on-site measurement.

Secondly, potentiometric ion-selective electrodes (ISEs), a kind of electrochemical ion sensors (i.e., potentiometric, amperometric, and conductometric), show a great advantage in the on-site measurement of phosphate due to their excellent analytical performance, simplicity of experiment and instrument, and low-cost [15]. An example worth mentioning comprises a commercial potentiometric Cu ISE of inner solution type equipped with pH and oxygen sensors for copper detection in freshwater. The results of the model were verified by potentiometric titration of a sample of freshwater using Cu, pH, and NH_3_ ISEs [16]. Over the last few years, all-solid-state potentiometric ISE have shown great potential in environmental water analysis [17]. All-solid-state ion-selective electrodes (ASS-ISEs), internal solution free, have the advantages of small sample volume, convenient maintenance, simple operation, and low-cost in an on-site measurement application, compared with conventional inner solution type ISEs [18]. The sensitive layers of solid phosphate ASS-ISEs are always cobalt, molybdenum, and a mixture of aluminum, aluminum phosphate, and copper powder [19]. Among these different metals, cobalt is a highly sensitive layer for phosphate ASS-ISE due to its high affinity. Xiao D. et al. prepared a new phosphate ion-sensitive electrode based on CoO layer and discussed the response mechanic in terms of the host-guest chemistry of a nonstoichiometric compound of CoO [20]. Zou Z.W. et al. developed a disposable microsensor for phosphate concentration measurement in aqueous solution based on fully integrated on-chip planar cobalt microelectrodes, Ag/AgCl reference electrodes, and microfluidic channels [21]. The research of solid phosphate ISEs mainly focuses on the use of metal-based materials to improve the performance, selectivity, and lifetime of these films.

The challenge now lies in using those sensors for on-site measurement and obtaining valuable chemical information directly in the field while minimizing or avoiding the need for sampling. Screen printing electrode (SPE) is a promising technology because of its simplicity, low-cost, high reproducibility, efficiency in large-scale production, and suitable for field-based phosphate analysis [21]. The distinctive cobalt oxide (CoO) group has shown a good selectivity towards different phosphate species (PO_4_^3−^, HPO_4_^2−^, H_2_PO_4_^−^), but the response mechanism is subject to some debates by being either mixed potential mechanism [22] or even Nernst potential [23]. An innovative, disposable cobalt-based phosphate sensor was fabricated using the SPE, at the Co/C ratio of 1:99, the cobalt-based phosphate sensor shows phosphate-selective potential response in the range of 10^−4^ to 10^−1^ mol/L, a slope of over 30 mV/decade in acidic solution (pH 4.5) for H_2_PO_4_^−^ [24], which have showed enormous potential for cost-effective and on-site analysis of wastewater samples. A potentiometric cobalt-based screen-printing sensor was fabricated by electroplating. The electroplating sensor performed linearly in the range of 10^−5^ to 10^−1^ mol/L and exhibited high sensitivity (−37.51 mV/decade) toward phosphate ions. The electrochemical performance of this sensor was fully examined to determine the interference of pH, various ions, and dissolved oxygen (DO) on the potential response [25]. Another phosphate sensor based on nickel oxide modified carbon SPE has been reported, which shows great potential for future scalable production and applications in portable real-time monitoring of phosphate ions in different precision agricultural and point-of-care diagnostic applications [26]. However, frequent recalibrations towards factors causing potential drifts, like pH and dissolved oxygen (DO), are still required by these sensors [27]. Total ionic strength adjustment buffer (TISAB) is widely used to shield the influence of interference factors. The addition of TISAB to the hydroponic solution will bring several disadvantages, including the need for complex operations and expensive chemicals, as well as causing pollution of the hydroponic system.

The nutrient concentration in hydroponic solutions is controlled by modulating only electrical conductivity (EC) and pH values at present [28,29]. Therefore, the closed hydroponic system still poses many challenges such as the imbalance in nutrient ratios caused by the lack or excess of some ions. ISEs array is a considered useful tool for determines multi-ions simultaneously in samples of complex hydroponic solutions. Artificial neural network (ANN) algorithm for the interpretation of the interferences was developed for prediction of NO_3_, K, Ca, and Mg ions in hydroponic solutions using an array of ISEs [30]. Authors combined the multivariate standard addition sampling technique with the deep kernel learning model for a six ISEs array to increase the prediction accuracy and precision of eight ions, including NO_3_^-^, NH_4_^+^, K^+^, Ca^2+^, Na^+^, Cl^−^, H_2_PO_4_^−^, and Mg^2+^ [31]. The absence of commercial phosphate ISEs limits the model’s better performance. Therefore, a buffer-free phosphate sensor with good consistency for batch production and popularization is urgently needed. At present, there is no precedent of directly using disposable phosphate electrode to determine phosphorus content in hydroponic solutions. The preparation process of electrodeposited cobalt-based phosphate ISE has the characteristics of simple manufacturing process, low-cost and suitable for mass production. Therefore, it is necessary to develop a Co-nanoparticles modified SPE for the determination of phosphate content in hydroponic solutions.

To address the aforementioned problems, this paper developed a disposable phosphate detection chip with good consistency for a hydroponic solution based on Co-nanoparticles modified carbon SPEs, which work without TISAB. A cobalt rod electrode is used as the substrate to scrutinize the effects of Co-nanoparticles on the detection of phosphate concentration. To minimize the disadvantages from Co-nanoparticles, the performance of the Co-nanoparticles modified disposable phosphate sensor was investigated. It is expected that the phosphate concentration can be measured quickly and accurately in the presence of strong cross-interference factors. So, the prediction model of phosphate in hydroponic solutions was established using SVR. Finally, we propose an example of its application in the direct detection of phosphate in hydroponic solution using the promising low-cost buffer-free sensor for on-site and rapid determination of phosphate concentration in hydroponic solutions.

## 2. Materials and Methods

### 2.1. Reagents and Apparatus

**Figure 1:**
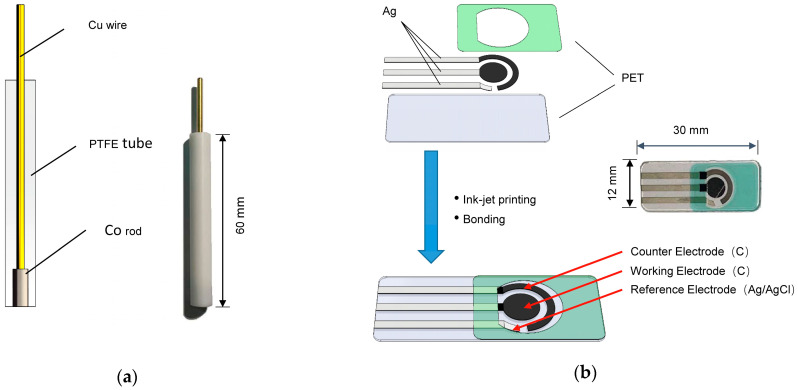
Schematic of the ion-selective electrodes (ISEs): (**a**) cobalt polytetrafluoroethylene electrode (Co_PTFE_ISE); (**b**) carbon screen-printed electrode (SPE).

Analytical reagents (AR) and deionized (DI) water were used for all solutions. Figure 1a shows a conventional cobalt polytetrafluoroethylene electrode (Co_PTFE_ISE), a cobalt rod (outer diameter: 4 mm) was inserted into a PTFE tube (inner diameter: 4 mm), which is used to demonstrate the effects of Co-nanoparticles on phosphate ISEs. The Co_PTFE_ISE surface area is 12.56 mm^2^. Figure 1b illustrates the schematic of the fabrication process of carbon SPEs. Carbon electrodes and wires onto a polyethylene terephthalate (PET) substrate were fabricated by an ink-jet printing method using inks containing different nanomaterials. The wires and connection terminals are made of Ag, the counter electrode and working electrode are made of carbon nanotube (CNT).The reference electrode is solidified Ag/AgCl.

An acidic electroplating solution for electrodeposition of the cobalt membrane was prepared. The basic components of the bath were cobalt sulfate heptahydrate (CoSO_4_·7H_2_O), sodium chloride (NaCl), and boric acid (H_3_BO_3_), with mass concentrations of 125, 17, and 30 g/L, respectively [24]. This acidic electroplating solution contains more cobalt, which can improve the speed of electrodeposition, a small amount of sodium chloride which can increase the solubility of the anode, and the boric acid which is used to adjust the pH value of the electroplating solution.

Standard phosphate solutions were prepared using a series of potassium dihydrogenphosphate (KH_2_PO_4_) dissolved in 0.05 mol/L potassium hydrogenphthalate (KHP) buffer at pH 4.0. To control the effect of DO and temperature on the response potential, the standard phosphate solutions for each experiment were prepared temporarily under the same conditions. The room temperature were kept at 25 °C throughout the experiment.

All electrochemical measurements and analyses were performed at 25 °C using an electrochemical workstation (CHI 660E, CH Instruments Inc., Austin, TX, USA). The microstructure of all the electrodes characterized by environmental scanning electron microscopy (SEM, Quanta 200FEG, FEI -Thermo Fisher Scientific, Waltham, MA, USA).

In order to inspect the performance of the phosphate detection chip in the on-site detection of a hydroponic solution, 100 hydroponic solutions containing different nutrients were prepared. The concentration range of each nutrient is 1 × 10^−1^–1 × 10^−5^ mol/L. The combination is carried out according to the principle of the orthogonal design.

### 2.2. Preparation of Modified Electrodes

Herein, the electrodeposited electrochemical cell consists of a three-electrode system, in which the corresponding carbon SPE and Co_PTFE_ISE are used as the working electrodes, the Ag/AgCl electrode as the reference electrode, and the platinum plate as the counter electrode (counter electrode of the carbon SPE is the corresponding carbon SPE). All the potentials in this paper are based on the Ag/AgCl electrode in a two electrodes system. The current density of the electrodeposition is 0.36 A/dm^2^. In the process of electrodeposition, the Co_PTFE_ISE is used as the cathodic electrode and the platinum electrode is used as the anodic electrode. The distance between the two electrodes is kept at 1 cm. For SPE, both the anodic electrode and the cathodic electrode are carbon electrodes. The working electrode of SPE is used as the cathode electrode to deposit Co-nanoparticles. During the process of electrodeposition, the electroplating solution was continuously stirred with a magnetic stirrer at the speed of 200 R/min to disperse the bubbles generated on the electrode.

### 2.3. Electrochemical Impedance Spectroscopy (EIS)

The EIS was measured by the method of alternating current (A.C.) impedance, which was carried out under the same conditions as for potentiometric measurements. EIS is an important means to study electrode process dynamics and surface phenomena in electrochemical measurement technology. Then, we can study the effects of Co-nanoparticles on phosphate ISE and the mechanism of electrode reaction. The initial potential is 5 mV. The characterized ISE had a 0.12566 cm^2^ surface area and an electrolytic cell filled with 1 × 10^−3^ mol/L KH_2_PO_4_ at pH 4.0. The EIS of the cobalt modified ISE was measurement in frequency range of 1–10^5^ Hz using the CHI 660E.

### 2.4. Behavior of Oxidized Co Electrodes in the Presence of Phosphate

**Figure 2:**
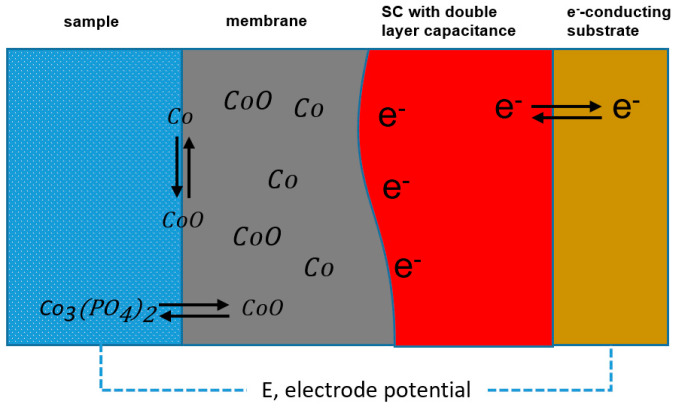
Schematic of oxidized Co electrodes in the presence of phosphate, oxidized Co electrode is an all-solid-state ISE based on a high-surface-area solid contact (SC) exhibiting a high double layer capacitance.

Co metal was introduced first as an electrode material for phosphate ion sensors in 1995 [20], due to it showed an excellent potentiometric response to phosphate ion. Meruva and Meyerhoff proposed that the potentiometric response resulted from the mixed potential of the slow oxidation of Co and the simultaneous reduction of oxygen and Co^2+^ [22]. The behavior of cobalt oxide electrode in the presence of phosphate can be described in Figure 2. At pH 4.0, acidic medium,

(1) $${2\mathrm{Co}+2H}_{2}O⇌{2\mathrm{CoO}+4H}^{+}{+4e}^{-}$$

(2) $$O_{2}{+2H}^{+}{+4e}^{-}⇌{2H}_{2}O$$

(3) $${2\mathrm{Co}+O}_{2}⇌2\mathrm{CoO}$$

(4) $${3\mathrm{CoO}+2H}_{2}{\mathrm{PO}}_{4}^{-}{+2H}^{+}⇌{\mathrm{Co}}_{3}{{(\mathrm{PO}}_{4})}_{2}{+3H}_{2}O$$

${\mathrm{Co}}_{3}{{(\mathrm{PO}}_{4})}_{2}$ is formed on the electrode surface in the presence of phosphate ions in the solution as the following reaction. Therefore, the cobalt electrode has a specific potential response to phosphate. From their study, it was observed that the response potential of phosphate ion selective electrode is affected by pH and DO.

### 2.5. Data Analyses

In order to ensure the repeatability and reproducibility of sensor data. Statistical differences among means were evaluated using Student’s t-test, ANOVA-single factor, and F-test at a 95% confidence interval (*p* < 0.05) [32,33].

## 3. Results and Discussion

### 3.1. The Effects of Co-Nanoparticles on Phosphate ISE

**Figure 3:**
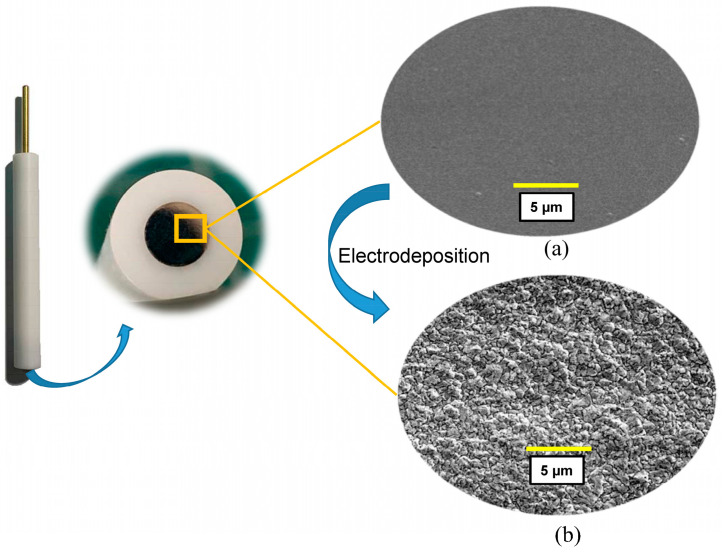
SEM diagram of the Co_PTFE_ISE surface before (**a**) and after (**b**) electrodeposition.

Using the conventional Co_PTFE_ISE as a substrate electrode, the effects of Co-nanoparticles on the detection of phosphate were analyzed. Figure 3 is the SEM diagram of the Co_PTFE_ISE surface before and after electrodeposition. Figure 3a shows the electrode surface after fine grinding with alumina powder with particle size of 1.0, 0.5, 0.3, and 0.05 μm, respectively. The Pristine_Co_PTFE_ISE surface polished by 50 nm alumina polishing powder is very smooth. Thus, a uniform flatness of the research electrode surface was ensured. Co-nanoparticles were deposited on the Pristine_Co_PTFE_ISE according to the method described above. The electrodeposition time was set to 20 min, so the 20_nCo_PTFE_ISE was obtained. Figure 3b clearly shows Co-nanoparticles deposited on the surface of 20_nCo_PTFE_ISE. The distribution of cobalt nano-particles is uniform and closely arranged.

#### 3.1.1. Effects on Calibration and Detection Limit (DL)

**Figure 4:**
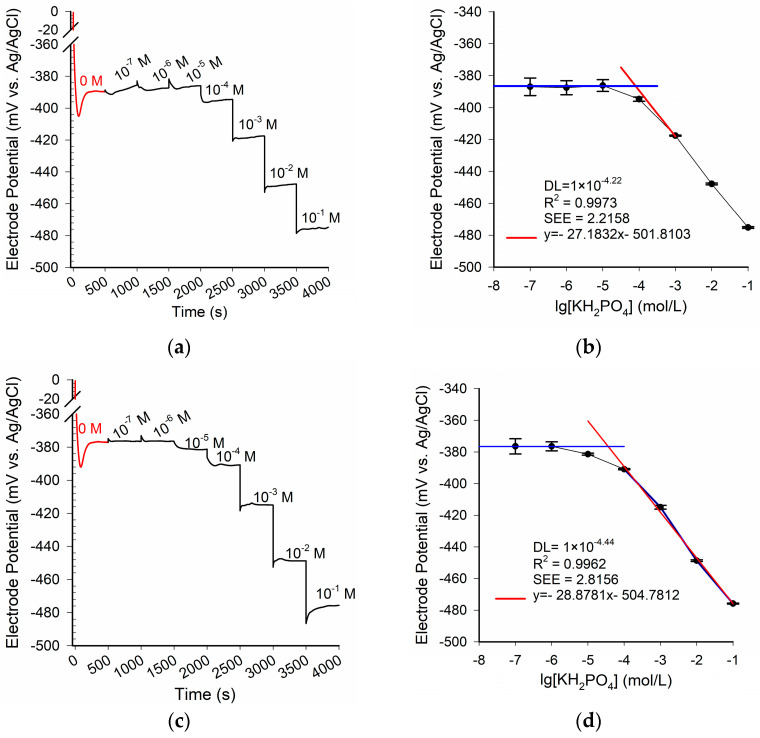
Potentiometric response curves of the phosphate sensor in different concentrations of KH_2_PO_4_: dynamic response (**a**) and calibration (**b**) for the Pristine_Co_PTFE_ISE; dynamic response (**c**) and calibration (**d**) for the 20_nCo_PTFE_ISE in the range of 1 × 10^−1^–1 × 10^−7^ mol/L.

For the purpose of investigating the effects of Co-nanoparticles on the phosphate ISE, the potential response curves of the pristine electrode and the modified electrode were measured as shown in Figure 4a,c. Pristine_Co_PTFE_ISE and 20_nCo_PTFE_ISE were placed in different concentrations standard phosphate solution for 500 s, the steady-state potential for the stepwise changes have been observed. While the two electrodes were placed in the buffer solution (KHP) without KH_2_PO_4_ (pH = 4.0, t = 0 s), the response to the step signal was rapid, and obvious overshoot appeared. In spite of this, it is optimistic that the overshoot and response time of the two electrodes in phosphate standard solution are significantly reduced (c[KH_2_PO_4_] > 0 mol/L). Co-nanoparticles did not bring significant changes to the response time and potential dynamic response characteristics of the electrode. As well, it is necessary to draw the linear calibration lines of the two electrodes as shown in Figure 4b,d. The dynamic response potential of the two electrodes were tested three times. The average of the steady-state response potential shown as the black dots in the Figure 4b,d. The linear regression equation (predictor) can be obtained by the least square method. The abscissa corresponding to the intersection point of the blue line and the red line is the exponential part of the DL value of the electrode. We can find that the detection limit (DL) of the modified Co-nanoparticles electrode is better. This indicates that the detection limit of the electrode can be enhanced by Co-nanoparticles. The coefficient of determination (R^2^) is the proportion of the variance in the dependent variable that is predictable from the independent variable. The standard error of the estimate (SEE) is a measure of the accuracy of linear regression predictor. In the best case, the modeled values exactly match the observed values, which results in R^2^ = 1, SEE = 0.

**Figure 5:**
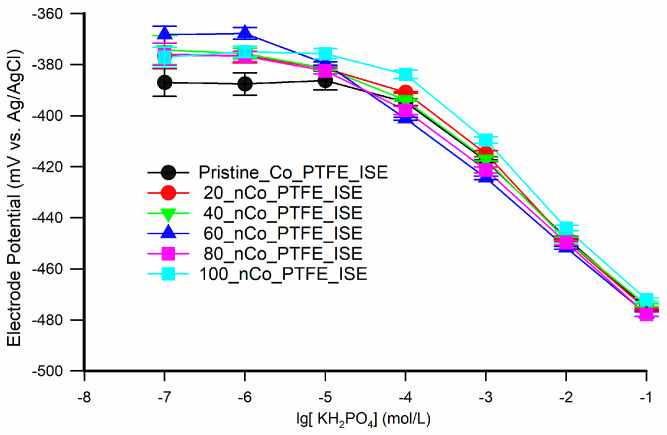
The response potentials of the nCo_ PTFE_ ISEs in the concentration range of 1 × 10^−1^–1 × 10^−7^ mol/L.

The coefficient of determination of the Pristine_Co_PTFE_ISE and the 20_nCo_PTFE_ISE are almost the same in the measuring range (linear range). The SEE of the 20_nCo_PTFE_ISE is slightly smaller than that of the Pristine_Co_PTFE_ISE in the linear range. The 20_nCo_PTFE_ISE has higher slope of linear regression equation (which can be regarded as sensitivity), but the performance improvement is not significant. Song et al. has verified by SEM and X-ray photoelectron spectroscopy that phosphate response at the cobalt surface was based on a change in the oxidation corrosion potential [24]. The reason, in fact, may be due to the short time of electrodeposition, the formation of Co-nanoparticles sensitive layer is too thin, and the thin cobalt layer can be corroded quickly. To investigate the influence of electrodeposition time on the optimization of electrode detection, the electrodes electrodeposited for 40, 60, 80, and 100 min were fabricated and analyzed according to the above method. The response potentials of the six electrodes in the concentration range of 1 × 10^−1^–1 × 10^−7^ mol/L are represented by dots of different colors in Figure 5.

The difference of response potential between the six electrodes is obvious in the low concentration phosphate standard solution (<1 × 10^−5^ mol/L). The response potential of the electrode modified by Co-nanoparticles is higher than that of the pristine electrode in low concentration phosphate solutions, which leads to the optimization of detection limit and linear range. The response potential of the modified electrode is not directly proportional to the electrodeposition time in the low concentration range of 1 × 10^−5^–1 × 10^−7^ mol/L. It can be found that the low concentration response potential of the 60_nCo_PTFE_ISE corresponding to blue is the highest.

**Figure 6:**
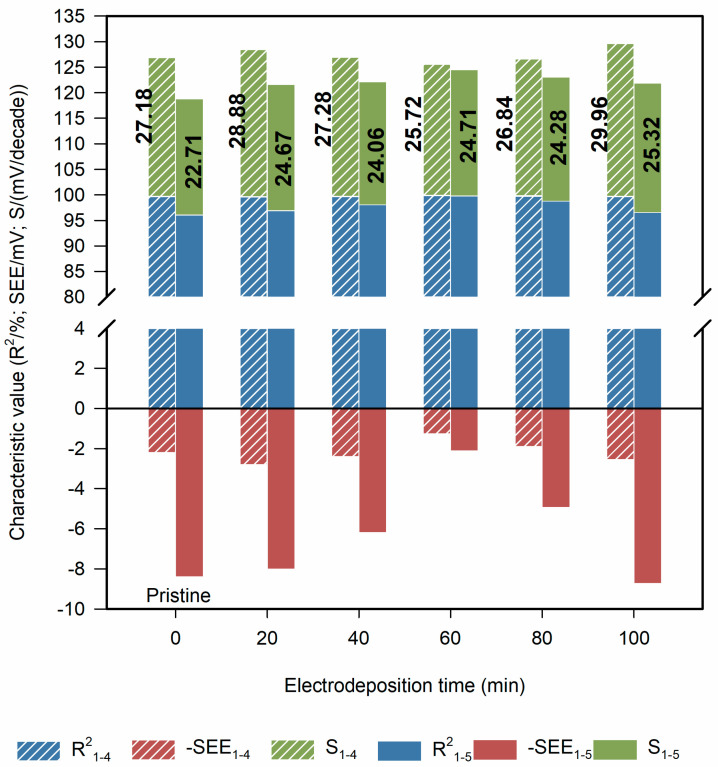
The performance evaluation of ISEs’ linear regression: R^2^ represents the determination coefficient (%); -SEE represents negative number of the SEE (mV); S represents the slope of the equation which is also sensitivity (mV/decade); the subscripts 1–4 indicate that the range of linear regression is 1 × 10^−1^–1 × 10^−4^ mol/L; the subscripts 1–5 indicate that the range of linear regression is 1 × 10^−1^–1 × 10^−5^ mol/L.

Figure 6 summarized the performance evaluation parameters of ISEs’ linear regression. The performance evaluation parameters of linear regression in different concentration ranges of 1 × 10^−1^–1 × 10^−4^ mol/L and 1 × 10^−1^–1 × 10^−5^ mol/L were investigated. Significant changes in performance parameters were not observed except for a slight difference in SEE. Moreover, in the concentration range of 1 × 10^−1^–1 × 10^−4^ mol/L, the advantage of the electrode modified by Co-nanoparticles is still not obvious, as we can see in the Table 1. Co-nanoparticles mainly affect the response potential of phosphate ISE at low concentration.

We can conclude that the response potential of the modified ISEs to low concentration phosphate (<1 × 10^−5^ mol/L) is increased; thus, the performance parameters of the linear regression in the concentration range of 1 × 10^−1^–1 × 10^−5^ mol/L are better than the pristine ISE. This phenomenon may be related to the higher specific surface area of Co-nanoparticles modified phosphate ISE. Therefore, more research is needed to identify this issue.

#### 3.1.2. Effect on Response Stability

**Figure 7:**
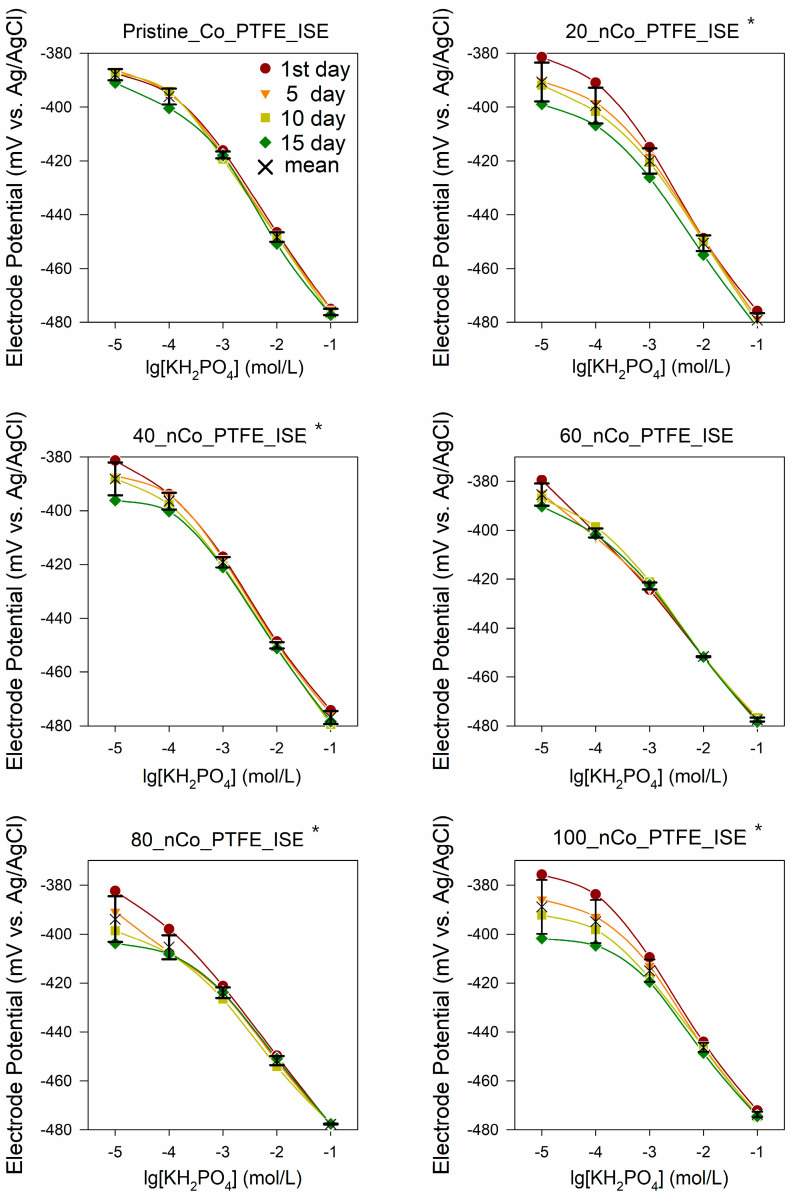
Potentiometric response curves of the phosphate sensor in different concentrations of KH_2_PO_4_ measured on the 1st, 5th, 10th, and 15th day; the “*” sign in the figure indicates that the time drift of the electrode is significantly different from that of the original electrode.

It is necessary to investigate its influence on the electrode stability. When each electrode was prepared, the dynamic potential response was measured on the 1st, 5th, 10th, and 15th day, as shown by different colored dots in Figure 7. After completing the daily test, washing the six electrodes with deionized water, and drying them with nitrogen gas after daily measurement. The stability studies have been carried out using the same electrode for the different days. The cross is the average response potential of the same concentration in the 15 days. The one-way ANOVA test was performed with significance defined as *p* < 0.05 to check whether the sensor-to-sensor potential drift variation was significant. The “*” sign in the figure indicates that the time drift of the electrode is significantly different from that of the original electrode. In the figure, we can also see that the response potential drift in the 15 days of 20_nCo_PTFE_ISE and 100_nCo_PTFE_ISE are easily observed. The potential drift of the Pristine_nCo_PTFE_ISE and 60_nCo_PTFE_ISE are relatively small.

The linearity of 60_nCo_PTFE_ISE is better. Response potential drift in 15 days is taken as the time drift of the electrode. Time drift of the phosphate ISEs in different concentrations of KH_2_PO_4_ as shown in Table 2. The 1 × 10^−5^ mol/L phosphate standard solution exceeded the minimum detection limit of the except for 60_nCo_PTFE_ISE, so they were not considered. When the electrodeposition time is more than 60 min, the potential drift of the electrode to phosphate of various concentrations increases obviously, which indicates that different electrodeposition time affects the distribution of Co-nanoparticles on the surface of the electrode, so that the detection range and stability of the electrode are different. The results show that the time drift of each electrode is large at low concentration and small at high concentration. This phenomenon is still related to the effect of Co-nanoparticles on the response potential of ISE at low concentration. Therefore, under some conditions, Co-nanoparticles will increase the time drift of ISE, which will reduce the stability of the electrode.

Overall, the time drift of the ISEs are increased to some extent by Co-nanoparticles. This may be due to the ablation of Co-nanoparticles during detection, which changes the characteristics of the initial surface of the optimized electrode. Different electrodeposition time directly affects the thickness of cobalt sensitive membrane. When the cobalt electrode contacts with phosphate solution, cobalt nanoparticles are corroded. Therefore, if the time of electrodeposition is too short, the substrate of the electrode may be exposed locally, resulting in serious time drift of the electrodes. When the electrodeposition time is too long, the electrodeposition layer on the electrode surface will be uneven, which will also lead to serious time drift [34,35]. Therefore, the electrodeposition time affects the characteristics of Co-nanoparticles on the electrode surface, which affects the properties of the electrode, so it is important to find the optimal electrodeposition time.

#### 3.1.3. Electrode Process Dynamics and Surface Phenomena

**Figure 8:**
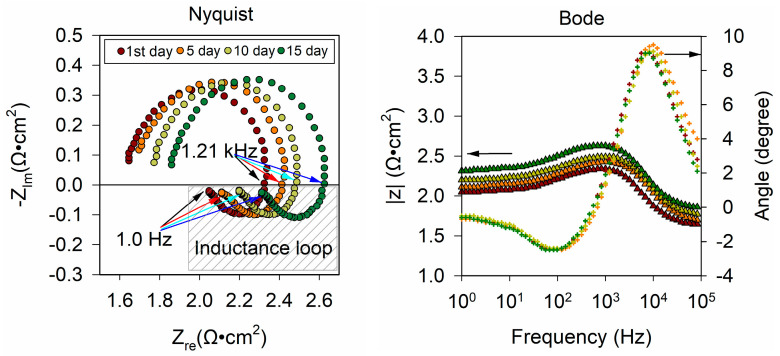
The Electrochemical Impedance Spectroscopy (EIS) of 60nCo_PTFE_ISE was measured on the 1st, 5th, 10th, and 15th day.

The electrochemical impedance spectroscopy (EIS) of 60_nCo_PTFE_ISE was measured in order to investigate the response mechanism of Co-nanoparticles modified ISEs. The EIS of 60_nCo_PTFE_ISE was measured on the 1st, 5th, 10th, and 15th day, as shown in the Figure 8. The nyquist plot is four curves drawn by dots of different colors with the real part of the impedance as the abscissa and the imaginary part of the impedance as the vertical axis. There are low-frequency inductance loop and depressed semicircle in all four curves. The ISE membrane/solid contact interface can be described as an asymmetrical electrical capacitor, it is the cause of inductance at low frequency. This indicates that the Co-nanoparticles modified phosphate ISE is an all-solid-state ISE based on a high-surface-area solid connect exhibiting a high double layer capacitance. In this case, the frequency characteristic of the double layer of the solid electrode is inconsistent with the capacitance (dispersion effect). The inhomogeneity of electric field in the double layer is related to the Co-nanoparticles on the electrode surface. This confirms the fact that the specific surface area of the sensitive film increases with the Co-nanoparticles. A constant phase element (CPE) is added to the equivalent circuit that describes the properties of the electrode system more accurately. There are two parameters to describe CPE (“Y_0_” and “n”). Since CPE is used to describe the physical quantity when the capacitance parameter deviates, Y_0_ always takes positive value as same as capacitance. The other parameter n is a dimensionless index. Q is the symbol of CPE, so its impedance can be calculated according to the following formula: Z_Q_ = 1/Y_0_·(jω)^−n^. From the abscissa of the high-frequency band, the solution resistance R_u_ of the electrode system cannot be overlooked. Each Bode plot consists of two curves, one is to describe the change of impedance modulus with frequency, called modulus diagram; the other is to describe the relationship between impedance phase angle and frequency, called phase diagram. At low and high frequencies, a straight line parallel to the abscissa corresponds to a phase angle less than 4° which is similar to the impedance of resistance R. There is an upward trend in the range of 10–10^4^ Hz, and then the downward trend is caused by the capacitor element. Therefore, there is a composite element (circuit description code: LR) composed of a resistor and an inductor in series.

**Figure 9:**
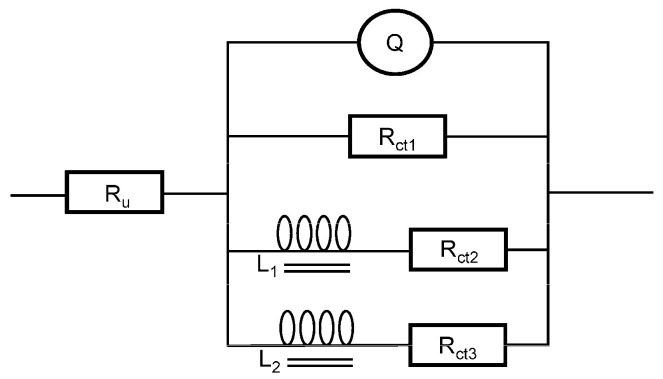
The equivalent circuit of the 60nCo_PTFE_ISE in the 1 × 10^−3^ mol/L KH_2_PO_4_.

Based on the preliminary analysis, the equivalent circuit of the electrode system is drawn and fitted, as shown in the Figure 9. The error of each parameter is required to be less than 10%, and the mean square of residual is less than 5.0 × 10^−5^. Where R_u_ is the ohmic resistance in solution, Q is the symbol of constant phase element (CPE), R_ct_ is charge transfer resistance and L is inductance. Hence, the circuit description code (CDC) of the circuit is R (QR (LR) (LR)).

The results of the equivalent circuit fitting are summarized in Table 3 and the maximum rate-of-change for each element in 15 days is calculated. The biggest rate-of-change is Y_0_, which reaches 94.57%, and the value decreases; that is, the impedance of the constant phase angle element increases. The value of n is always between 0.5 and 1, so Q is ordinarily considered that the Q element has the property of capacitance. In addition, the more n deviates from 1, the stronger the “dispersion effect” of the electrode surface will be. The n increases slightly with time, which indicates that the number of Co-nanoparticles attached to the electrode surface decreases. That is consistent with Lei’s conclusion that the phosphate response of the cobalt surface based on a change of oxidation corrosion potential [22]. Electrochemical impedance spectroscopy (EIS) showed that the corrosion of Co-nanoparticles on the electrode surface was changed by the corrosion between cobalt and phosphate, which would affect the response potential and lead to more potential drift. Moreover, the increase of inductance in Faraday impedance is larger. The rate-of-change for charge transfer resistance is slightly smaller. The increasing charge transfer resistance means that Co_3_(PO_4_)_2_ is formed on the electrode surface in the presence of phosphate ions in the solution.

From the discussion, one may conclude that Co-nanoparticles improve the detection performance of phosphate by improving the specific surface area of the electrode, especially the detection ability of low concentration. However, the corrosion of cobalt is more rapid, so there is a serious time drift of the electrode, which finally brings instability to the electrode. Accordingly, the optimization of phosphate ISE by Co-nanoparticles is limited to the initial state of the electrode. Under the condition of electrodeposition in this paper, the electrodeposition time of about 60 min is more suitable.

### 3.2. Disposable Sensor Based on Co-Nanoparticles

#### 3.2.1. Proper Electrodeposition Time of SPEs

**Figure 10:**
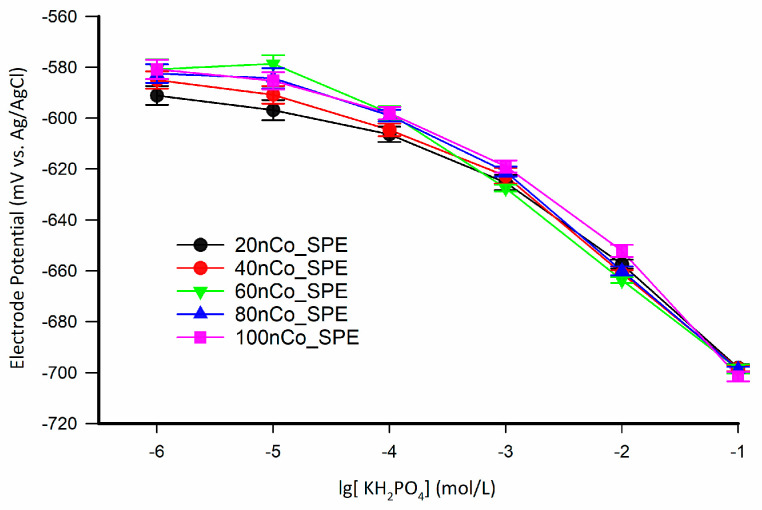
The response potentials of the nCo_SPEs in the concentration range of 1 × 10^−1^–1 × 10^−6^ mol/L.

Disposable phosphate detection chip can be used to minimize the influence of the time drift. The disposable chip should have the characteristics of low-cost and batch production. To verify the influence of electrodeposition time on the optimization of electrode detection on the SPE and the electrodeposition time for manufacturing a phosphate detection chip is acceptable, the SPEs electrodeposited for 20, 40, 60, 80, and 100 min were fabricated. The response potentials of the nCo_SPEs in the concentration range of 1 × 10^−1^–1 × 10^−6^ mol/L as shown in Figure 10. The detection limits of these electrodes for standard phosphate solutions are 10^−4^ mol/L. There are step response potentials at different concentrations. It seems that the difference caused by electrodeposition time is not visible, but the potential response at low concentrations can be detectable. To investigate the influence of deposition time on the electrode more clearly, the performance evaluation parameters of linear regression in concentration range of 1 × 10^−1^–1 × 10^−4^ mol/L were investigated as shown in Table 4. Although the sensitivity of 60nCo_SPE is not the highest, it has a better coefficient of determination, a wider linear range, and a lower standard deviation. Following the above conclusion, the optimal electrodeposition time under the electrodeposition conditions described in this paper is about 60 min for SPE. The proper electrodeposition time is necessary for the fabrication of a phosphate ISE with good initial performance.

**Figure 11:**
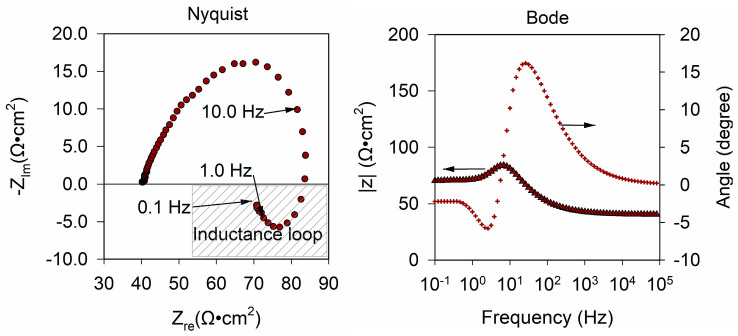
The EIS of 60nCo_SPE was measured on the 1st day.

In order to compare the two electrodes more comprehensively, we added the EIS of disposable SPE as shown in Figure 11. Through the equivalent circuit fitting, we get the same equivalent circuit as Figure 9. From the fitting results in Table 5, the resistance of SPE is higher than that of PTFE_ISE because the substrate of SPE is a thin carbon coating. The smaller n indicates that the surface of SPE is rougher. The components in the equivalent circuit of the two electrodes are only different in numerical value. We found that the charge transfer mechanism of SPE is consistent with that of traditional electrode.

#### 3.2.2. Calibration and Consistency Test of nCo_SPE

The electrodeposition time is set at 60 min to make the disposable phosphate detection chips (nCo_SPE). The consistency of the phosphate detection chip mainly determines the accuracy of the sensor. Because it is difficult to calibrate each phosphate detection chip. For phosphate detection chip, the response potential of each phosphate detection chip under the same manufacturing process to the same signal source (the same phosphate concentration) should have a tiny difference.

**Figure 12:**
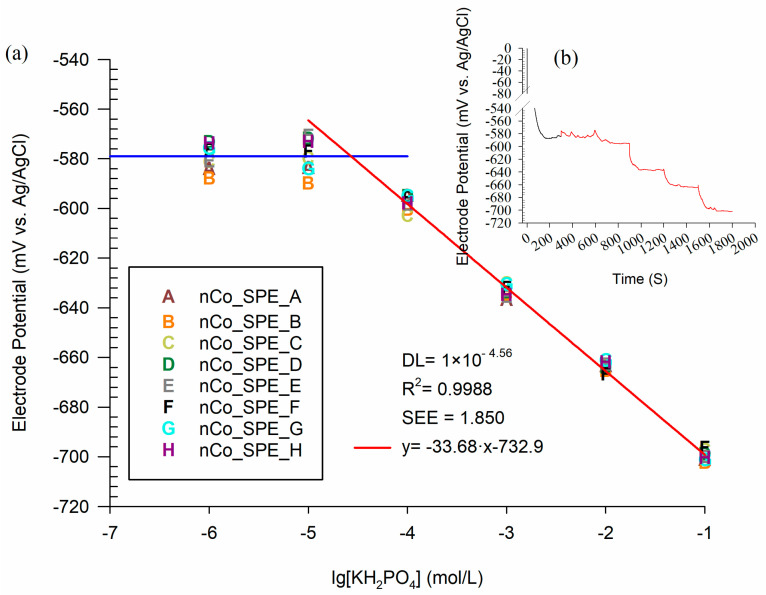
Calibration (**a**) for the low-cost phosphate detection chips in the range of 10^−1^–10^−7^ mol/L, (**b**) potentiometric response curve of nCo_SPE_A.

Eight low-cost disposable phosphate detection chips were made to investigate the consistency of the manufacturing process. The potential response of the eight SPEs is shown as the letter in Figure 12a, and the dynamic potential response curve of nCo_SPE_A is shown in Figure 12b. While the concentration of phosphate is more than 10^−5^ mol/L, the dispersion of the response potential of the chip is small. The results from the ANOVA-single factor analysis revealed that there was no significant difference among the eight SPEs (*p* > 0.05). This means that the electrode has good stability when the phosphate concentration is within the detection range. The calibration curve of the arithmetic mean of the response potentials of the eight electrodes is shown in the red curve in Figure 12a. The linear regression showed that the phosphate sensor had good linearity (R^2^ = 0.9988), low error (SEE = 1.85 mV) and high sensitivity (S = 33.68 mV/decade).

To quantify the evaluation of the consistency among the eight electrodes, the range, standard deviation, arithmetic mean, and coefficient of variation of the response potentials of the eight electrodes in phosphate solutions of different concentrations are summarized in Table 6. Range (statistics), the difference between the highest and the lowest values in a set. Since it only depends on two of the observations, it is most useful in representing the dispersion of small data sets. Standard deviation is a measure of the amount of variation or dispersion of a set of values [36]. The coefficient of variation (CV, %) is defined as the ratio of the standard deviation to the mean. The CV is widely used in analytical chemistry to express the precision and repeatability of an assay. It is conventional used in fields such as engineering or physics when doing quality assurance studies. In the linear range, the maximum coefficient of variation of the sensor is only 0.4992%. Hence, the phosphate detection chip has a good consistency.

#### 3.2.3. Response Time and Lifetime of the nCo_SPE

**Figure 13:**
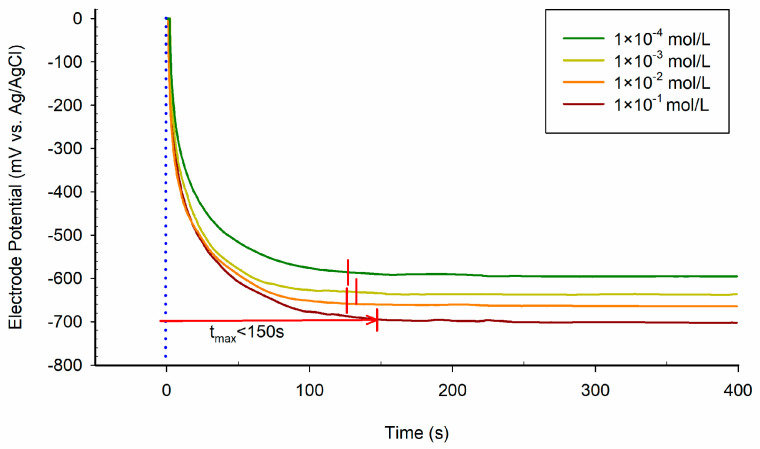
Response time of the low-cost phosphate detection chip in different concentrations of KH_2_PO_4_.

Response time is also an important indicator of the dynamic performance of the phosphate detection chip. The time from the contact signal source to the steady-state of the phosphate detection chip is regarded as the response time. The response time at different concentrations is shown in Figure 13. The maximum response time is less than 150 s in phosphate standard solution with a concentration of 1 × 10^−1^ mol/L. Therefore, the phosphate detection chip has an acceptable response time of about 150 s.

**Figure 14:**
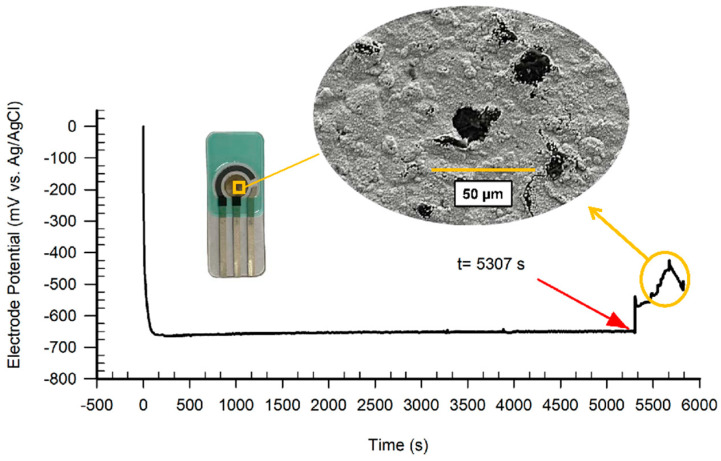
The long-term potentiometric response of the phosphate detection chip in the 1 × 10^−2^ mol/L KH_2_PO_4_.

To investigate the continuous detection life of the low-cost disposable phosphate detection chip. The long-term potentiometric response of the phosphate detection chip is measured as shown in Figure 14. The potential signal of the phosphate detection chip began to fluctuate slightly after 3000 s. Then, a large signal distortion appeared at 5307 s. This indicates that the sensor can maintain a stable signal for at least 3000 s even in a high concentration (10^−2^ mol/L) of the standard phosphoric acid solution. A 5307 s reliable signal can be provided continuously. Co-nanoparticles on the surface of the nCo_SPE with signal distortion have been corroded and resulting in the exposure of the electrode substrate.

In this section, we got a phosphate determining chip with good linearity, low error, high sensitivity, good consistency, and acceptable response time and lifetime. A phosphate sensor with excellent linearity, low error, and sensitivity mean that the sensor can accurately detect the concentration of phosphate under appropriate conditions. The consistency of the mass-produced disposable detection chip determines the popularization of the sensor. The good consistency of disposable sensors means that different chips do not need to be calibrated one by one. The same batch of chips use the same calibration predictor, so the availability of sensors is greatly improved. The acceptable response time and lifetime are the preconditions for the disposable phosphate determining chip to be utilized for on-site and rapid detection.

### 3.3. Determination of Phosphate Concentration in Hydroponic Solutions

It is crucial to investigate the practical application of the sensor in a hydroponic solution. Certainly, the phosphate detection chip is not a specific electrode. In addition to the response to phosphate ion, it also has more or less response to some other ions and will be affected by DO and pH value of the solution [24]. This interference factor exists more or less on all ISEs, which is difficult to avoid entirely. In hydroponic solutions, there are strong cross-interference between different nutrient ions and other interfering factors such as temperature and DO [37]. To determine phosphate concentration in hydroponic solutions directly, TISAB needs to be dropped into the solution. However, it is impractical to drop TISAB into hydroponic solutions, which will seriously affect the environment of crop growth.

**Figure 15:**
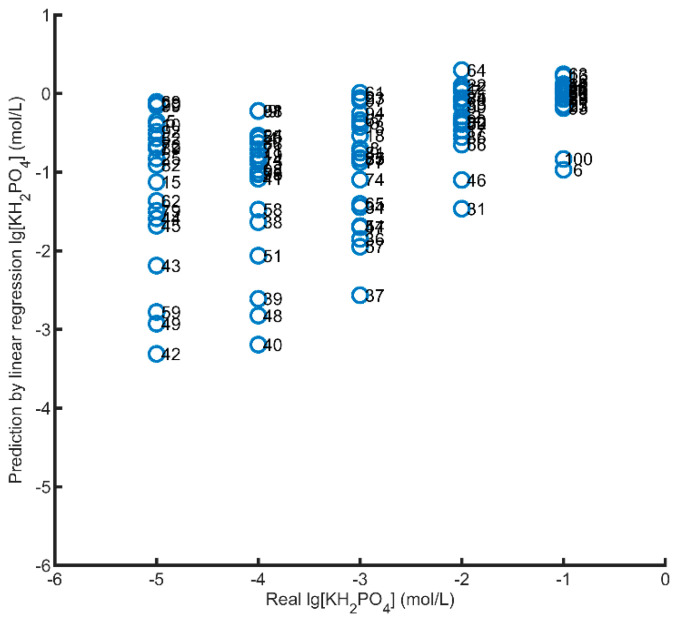
Real value vs. Predicted value predicted by the linear regression; Arabic numerals are the sample numbers of hydroponic solutions.

In order to direct the determination of ions concentration in the real samples without the necessity of eliminating the interferences, multivariate nonlinear regression methods were often used to establish prediction model [38,39,40,41,42,43,44,45,46,47]. In this work, support vector machine regression (SVR) is used to eliminate the influence of these interference factors. It is worth mentioning that the phosphate detection chip with good consistency can greatly improve the generalization ability of prediction model. The sensor array consists of potassium, ammonium, and nitrate ion selective electrodes (potential response signals) and DO, pH, and temperature electrodes (voltage signals). The outputs of the sensors array is introduced to establish the support vector regression prediction model. The response signals (100 × 7 matrix) of 100 hydroponic solution samples were tested. The concentrations of phosphate were directly inversed by the linear regression predictor, which compared with the real concentration corresponding to the sample, as shown in Figure 15. Under the interference of many factors, the prediction result of linear regression has a large deviation with the real value. Without the use of any buffer, the measurement results are distorted and unreliable.

**Figure 16:**
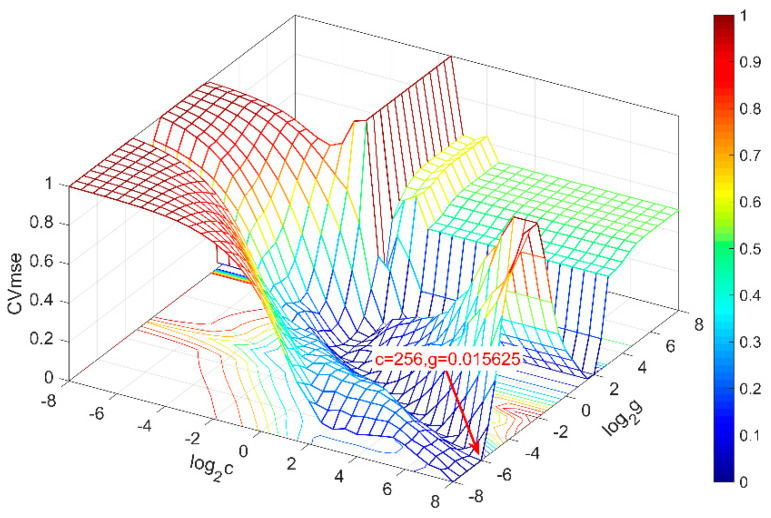
3D view of SVR parameter selection results (GridSearch), the best *c* = 256, *g* = 0.015625, MSE = 0.23592.

Ten samples were randomly selected as a test set and other hydroponic solutions as a training set to establish an SVR prediction model for phosphate detection chip. The parameters of support vector regression (*c* is the penalty coefficient, *g* is the radius of kernel function) is selected by the grid search method as shown in Figure 16. The mean square error (MSE) is a measure of the quality of a prediction model, which is always non-negative, and values closer to zero are better. Taking *c* = 256, *g* = 0.01, the minimum mean square deviation of the calibration set is 0.2359. Such a prediction model for the phosphate detection chip is established.

**Figure 17:**
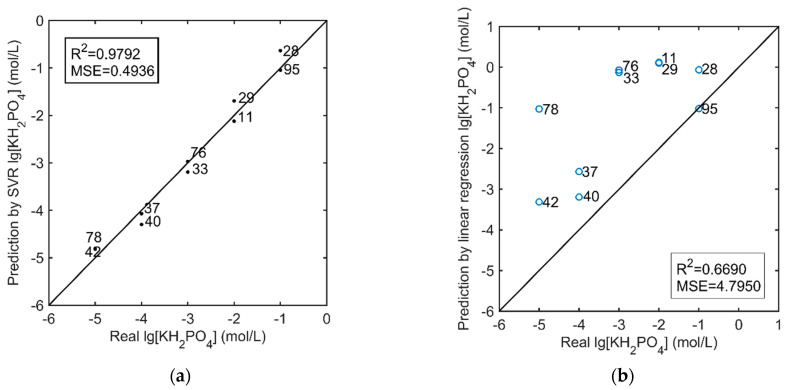
Real value vs. Predicted value for the (**a**) prediction by the SVR, and (**b**) prediction by the linear regression; Arabic numerals are the sample number of the validation set.

The test set was used to examine the prediction effect and the generalization ability of the model. Figure 17a shows the correlation between SVR predicted values and real values of hydroponic solutions in the test set. Compared to the 1:1 line, which is represented as one continuous line, it is evident that predictions were satisfactory for the phosphates ions in hydroponic solution. The correlation between SVR predicted value and real value is very good (R^2^ = 0.9792). The MSE of the test set is acceptable, but there is still scope for optimization (MSE = 0.4936). Compared with the prediction results inversed by the linear regression, as shown in Figure 17b, the predicted values have seriously deviated under the influence of multiple factors. The measurement results eliminate the distortion caused by critical interference. It indicates that the phosphate detection chip can measure the concentration of phosphate in hydroponic solutions on-site and rapidly.

In this paper, the consistency of the phosphate detection chip for the hydroponic solution based on Co-nanoparticles modified screen-printed electrode is examined to ensure that it can model generalization. Next, regarding the introduction of the calibration electrode and sensor signal, the prediction model was established by using SVR, so that the phosphate detection chip could determinate of phosphate concentration in the hydroponic solution directly. However, there is no phosphate specific composite multi-electrode chip. The introduction of the calibration electrode brings some troubles to the use of the phosphate detection chip. An effective method is to make a multi-channel printed electrode array with the corresponding electrode of specific interference factors and phosphate directly to solve the inconvenience caused by the introduction of the correction electrode [48]. It is important that the phosphate detection chip based on a multi-channel printed electrode also needs good linearity and sensor consistency.

## 4. Conclusions

This paper concerns the optimization of phosphate ion selective electrodes modified by Co-nanoparticles and proposes an example of its direct application in the detection of phosphate in hydroponic solutions. Firstly, the effects of Co-nanoparticles on the detection performance of phosphate ISEs are studied. Co-nanoparticles increased the response potential of phosphate ISE in low concentration. Through the corrosion of Co-nanoparticles resulted in a severe time drift of ISE, the initial performance of phosphate ISE was improved by Co-nanoparticles. Moreover, the electrodeposition time had a significant effect on the optimization effect.

Then, the disposable phosphate detection chip was fabricated based on SPE to avoid the time drift from the corrosion of Co-nanoparticles. The phosphate detection chip with the same modification process has good sensitivity (33 mV/decade) in the linear range (10^−1^–10^−4.56^ mol/L). The chip has a satisfactory consistency with the coefficient of variation less than 0.5%.

Finally, six correction signals were supplemented to establish the prediction model based on SVR so that the phosphate detection chip has the ability to determine phosphate concentration in hydroponic solutions on-site and rapidly, and the determination coefficient of the prediction model reached 0.9792.

In summary, this paper argued that Co-nanoparticles modified SPE is a promising disposable phosphate detection sensor. For the first time, a disposable phosphate electrode was used to directly measure the phosphate concentration in hydroponic solutions. The interference factors such as dissolved oxygen, pH, and temperature were corrected. The preparation process of the disposable phosphate electrode has the characteristics of a simple process, low-cost, and suitable for mass production. The future research will focus on developing a phosphate detection chip with multi-channel correcting electrodes.

## Figures and Tables

**Table 1:** Linear Response Range of Electrodes.

T (min) ^1^	Linear Response Range (mol/L)
0 (Pristine)	1 × 10^−1^–1 × 10^−4.22^
20	1 × 10^−1^–1 × 10^−4.44^
40	1 × 10^−1^–1 × 10^−4.64^
60	1 × 10^−1^–1 × 10^−5.29^
80	1 × 10^−1^–1 × 10^−4.76^
100	1 × 10^−1^–1 × 10^−4.25^

^1^ Electrodeposition time.

**Table 2:** Time Drift of The Phosphate Sensors.

T (min)	Time Drift (mV) ^1^				
	1 × 10^−1^ mol/L	1 × 10^−2^ mol/L	1 × 10^−3^ mol/L	1 × 10^−4^ mol/L	1 × 10^−5^ mol/L
0 (Pristine)	2.1332	4.1910	3.1183	6.3274	-- ^2^
20	5.9685	6.1201	11.1566	15.9366	-- ^2^
40	5.1717	2.5115	4.0082	6.4033	-- ^2^
60	1.7931	0.3528	3.3329	4.3471	10.7320
80	0.4466	4.3878	5.2956	10.2450	-- ^2^
100	2.5064	4.4734	9.9574	20.9314	-- ^2^

^1^ Response potential drift in 15 days (Time drift); ^2^ The value is not analyzed because the detection limit of the electrode not reached 1 × 10^−5^ mol/L.

**Table 3:** The Results of The Equivalent Circuit Fitting.

Symbol	1st Day	5th Day	10th Day	15th Day	Roc (%) ^1^
R_u_ (Ω·cm^2^)	1.612	1.648	1.748	1.844	14.39
Q-Y_o_(E-5·Ω^−1^·cm^−2^·s^−n^)	150.5	13.18	8.164	8.685	94.57
Q-n (1)	0.8618	0.8424	0.8961	0.9014	6.84
R_ct1_ (Ω·cm^2^)	0.7913	0.8554	0.7902	0.8261	8.23
L_1_ (E-3·H·cm^2^)	1.592	1.375	2.074	2.804	89.76
R_ct2_ (Ω·cm^2^)	4.570	4.673	4.837	4.753	5.84
L_2_ (E-3·H·cm^2^)	4.838	4.440	5.164	6.740	47.54
R_ct3_ (Ω·cm^2^)	1.606	1.869	1.798	1.855	16.38

^1^ The maximum rate of change (%) obtained by dividing the maximum change during the test by the initial value.

**Table 4:** The Performance Evaluation Parameters of Linear Regression.

T (min)	R^2^ (%) ^1^	SEE (mol/L) ^2^	S (mol/L) ^3^	DL (mol/L) ^4^
20	97.53	7.7335	30.7227	1 × 10^−4.22^
40	97.79	7.5856	31.9330	1 × 10^−4.33^
60	99.82	2.2798	33.7913	1 × 10^−4.49^
80	98.54	6.5123	33.8250	1 × 10^−4.31^
100	96.68	10.0734	34.3724	1 × 10^−4.23^

^1^ The coefficient of determination; ^2^ The standard error of the estimate; ^3^ Sensitivity; ^4^ Detection Limit.

**Table 5:** The Equivalent Circuit Fitting of 60nCo_PTFE_ISE and 60nCo_SPE.

Symbol	60nCo_PTFE_ISE	60nCo_SPE
R_u_ (Ω·cm^2^)	1.612	40.29
Q-Y_o_ (E-5·Ω^−1^·cm^−2^·s^−n^)	150.5	84.26
Q-n (1)	0.8618	0.6371
R_ct1_ (Ω·cm^2^)	0.7913	64.51
L_1_ (E-3·H·cm^2^)	1.592	3313
R_ct2_ (Ω·cm^2^)	4.570	19.89
L_2_ (E-3·H·cm^2^)	4.838	3569
R_ct3_ (Ω·cm^2^)	1.606	60.33

**Table 6:** The Consistency of Phosphate Detection Chip.

Lg[KH_2_PO_4_] (mol/L)	Range (mV) ^1^	SD ^2^	AM (mV) ^3^	CV (%) ^4^
−1	5.8930	2.0454	−699.8479	0.2922
−2	5.6374	1.7999	−663.7935	0.2711
−3	6.6292	2.3993	−633.6228	0.3786
−4	8.0817	2.9838	−597.6359	0.4992
−5	19.0907	6.7715	−578.8720	1.1697
−6	14.6025	5.2141	−579.2224	0.9002

^1^ Range of the difference between the highest and the lowest values in same concentration; ^2^ Standard deviation is a measure of the amount of variation or dispersion of a set of values; ^3^ Arithmetic mean; ^4^ The coefficient of variation.

## Data Availability

Not applicable.
